# How should we interpret conclusions of TKI-stopping studies

**DOI:** 10.1038/s41375-023-02002-y

**Published:** 2023-08-25

**Authors:** Robert Peter Gale, Junren Chen

**Affiliations:** 1 Centre for Haematology, Department of Immunology and Inflammation, Imperial College of Science, Technology and Medicine, London, UK; 2 State Key Laboratory of Experimental Hematology, National Clinical Research Center for Blood Diseases, Haihe Laboratory of Cell Ecosystem, Institute of Hematology & Blood Diseases Hospital, Chinese Academy of Medical Sciences & Peking Union Medical College, Tianjin, China

**Keywords:** Cancer, Diseases

*TKI-stopping studies are like the New Yorker magazine; Highly admired but rarely read in detail*

For most physicians and people with CML the best outcome of tyrosine kinase-inhibitor (TKI)-therapy outcome is achieving therapy-free remission (TFR).^1^ The metric for achieving TFR is complex and needs to consider co-variates such as depth and duration of response and other co-variates known, unknown but knowable (*latent*) or perhaps unknowable. Chance may also be important.

There have been several studies analyzing relationships between depth and duration of TKI-therapy and likelihood of achieving TFR [For example, 1–9]. Amongst these the EURO-SKI is the largest. The study reported the longer someone received TKI-therapy and the longer their interval of deep molecular response (DMR), the greater their likelihood of achieving TFR. These correlations, although correct for the study subjects, are widely- and incorrectly-interpreted to indicate someone’s likelihood of achieving TFR is increased by continuing them on TKI-therapy and can be generalized to people on TKI-therapy unlike the study subjects. We explain why this interpretation is wrong or superficial or sometimes detrimental to people receiving TKI-therapy. Although we focus on the EURO-SKI study our critique applies to other TKI-stopping studies.

## Study population

The EURO-SKI study population was recruited from 61 European centers in 11 countries. 868 people were screened, 821 enrolled, 758 (92%) constituted the study cohort but only the first evaluable 200 (26%) were the basis of the interim analysis in the article. (Additional analyses are planned.) Composition of the study population raises several issues of concern the 1st of which is representativeness and generalizability. There is no indication centers were audited to ensure the screened population included all consecutive potentially eligible subjects raising the possibility of selection bias. Might there have been a bias to include subjects with the longest TKI-therapy intervals or longest interval of DMR knowing the trial involved stopping therapy? We cannot know this from the EURO-SKI data. We also don’t know if the study-cohort from European centers is representative of the universe of people with chronic phase CML on TKI-therapy in whom TKI-stopping is considered in the 11 study countries or, more importantly, elsewhere such as North America where therapy outcomes lag those in Germany for example [10].

Screening of potential subjects was from what was an observational database with unavoidable limitations and biases we discuss elsewhere [11,12]. Failure to pass screening could result from any of several reasons: (1) a potentially eligible subject declined to participate in the study; (2) a subject did not meet ELN response-criteria and therapy targets operational when they were treated and which changed during the >10 interval over which potential subjects were screened; (3) TKI-therapy was discontinued before start of the screening interval because of adverse events, cost, non-compliance or patient or physician preferences; and (4) the potential subject had a DMR but it was lost at the time of screening. Because of these potential biases we should not assume the study cohort reflects people with an average response to TKI-therapy but rather the *best* response, namely those who had remained on TKI-therapy with a DMR in good compliance without adverse events. Some estimate eligibility of the study cohort is less than one-half of everyone starting TKI-therapy [13].

To see how unlike the study-cohort is to contemporary persons with chronic phase CML we need to look at Table 1 of the EURO-SKI report [1]. 52 percent of subjects received therapy before starting TKI-therapy, often with hydroxyurea (45%). Importantly, median interval on TKI-therapy was 7.7 years (Interquartile Range [IQR], 5.1–10.4 years) and median duration of TKI-therapy was 7.5 years (5–9.9 years). In total, 25 percent of subjects were on TKI-therapy >10 years and only a quarter of subjects discontinued TKI after 3–5 years. Namely, the study population is not representative of people with chronic phase CML begun on therapy in the last 3–5 years.

## Survivorship bias

A 2nd concern is that in contrast to most prospective trials the study population was heterogeneous; some subjects entered the study population after 1 year of a deep molecular response (DMR) whereas others entered after 2, 4, 6 or 8 years of DMR. Obviously, subjects with an 8-year DMR differ from those with a 1-year DMR in that the 8-year cohort excluded everyone who had a 1-year DMR but subsequently *failed* for ≥1 of the reasons we discussed above.

Given the study-design it is unsurprising someone receiving and responding well to TKI-therapy for a prolonged interval might have a greater likelihood of successfully achieving TFR compared with someone receiving it for only a brief interval, responding to it poorly or both. This is a so-called: *self-fulfilling prophesy*. To illustrate, imagine a marathon race with 1000 people at the starting line. The starting gun goes off and 2 h later a TV camera man stationed at km 30 and unaware of the number of race entrants counts 200 runners who have passed him. Suppose he later learns 100 people successfully cross the finish line (i.e. 10% of race entrants). He would be wrong if he calculates 50 percent of racers (i.e. 100 out of 200) finish the race. In reality, 800 entrants quit before km 30 and the camera man observes only the best runners. Were the camera man to advise potential marathon runners: *All you need to do is run to km 30 and your chance of finishing the race will increase to 5-fold compared with when you started*. He would, of course, be giving a ridiculous and ineffective *prescriptive* suggestion. The important point is the population at km 30 is not representative of the starting population.

## Performance metrics and benchmarks

If we had performance data on all the race starters and finishers we could try to identify co-variates correlated with likelihood of finishing the race. Next we would typically develop a *predictive* (not *prescriptive*) model to identify finishers before the race started. A last step is to assess model accuracy by calculating the C- or Concordance statistic (equivalent to the area under the receiver-operator characteristic curve [AUROC]). This series of steps was not done nor *doable* in the EURO-SKI report.

But the EURO-SKI report would be hard to interpret even if data on everyone at the starting line were available. In the marathon example all runners start the race at the same time whereas in the EURO-SKI dataset runners start at different times (*i.e*. with different durations of TKI-therapy before DMR *etc*.). We also don’t know how many runners planned to enter the marathon but never made it to the starting line *(i.e*. never achieving DMR). These issues should influence one’s interpretation of conclusions of the EURO-SKI trial and other TKI-stopping studies.

It is important to note the EURO-SKI study conclusions regarding *predictive* co-variates apply only to persons initially treated with imatinib which is less than one-half of current people with CML. One of the main conclusions of the EURO-SKI report was to reject the *null hypothesis* 6-month molecular relapse-free survival is ≥40% but this hypothesis was based on an earlier STIM cohort (2007–2009) treated only with imatinib for a median of 4.2 years, whereas in EURO-SKI although 94 percent received imatinib as initial therapy many later received nilotinib or dasatinib [14]. Also, median duration of TKI-therapy was 3.3 years longer than in the STIM cohort. Therefore, rejecting this *null hypothesis* in the EURO-SKI could be explained by superiority of 2^nd^-generation TKIs over imatinib, not necessarily the benefit of longer TKI-therapy.

## Association versus causation

The authours of the EURO-SKI report stated that in the training dataset (limited to subjects receiving imatinib) “*longer treatment duration …and longer deep molecular response duration were****associated*** *with an increasing probability of MMR [major molecular response] maintenance* ***at 6 months****”* (Bolding ours). This is an appropriate conclusion for this study-design. For what we discussed above it should be clear the correlation between duration of TKI-therapy in the EURO-SKI report is an *association*, not *causation*. Put otherwise, it is incorrect to assume someone with a specific duration of DMR on TKI-therapy has a greater likelihood of achieving TFR if they continue therapy. There are two reasons for this: 1st, that person may have already reached his/her maximal likelihood of TFR success in which case continuing therapy cannot improve the likelihood of success (but can cause harm); and 2nd, there is no guarantee DMR will be maintained if TKI-therapy is extended (recall potential subjects who lost their DMR on TKI-therapy were excluded from the EURO-SKI study).

So what do the EURO-SKI data tell us? They tell us duration of TKI-therapy and duration of DMR (confounded co-variates) are a useful *predictive* test of the likelihood achieving TFR. What they do not tell us is that this greater likelihood is the result of prolonging TKI-therapy.

Let’s consider how the EURO-SKI data are being incorrectly interpreted by some physicians treating CML. 1st, some assume the EURO-SKI data guide TKI-therapy in general, even though their conclusion applies only to subjects receiving imatinib as a first-line TKI and should not be assumed to apply to people receiving other TKIs initially. 2nd, some wrongly assume the EURO-TKI data prove prolonging TKI-therapy improves likelihood of achieving TFR. Finally, some do not realize the TFR success point in the reported interim analysis was at 6 months after stopping; longer term outcomes are unknown although most molecular relapse occur in this interval.

## Can we determine the best duration of TKI-therapy for TFR success?

**Fig. 1:**
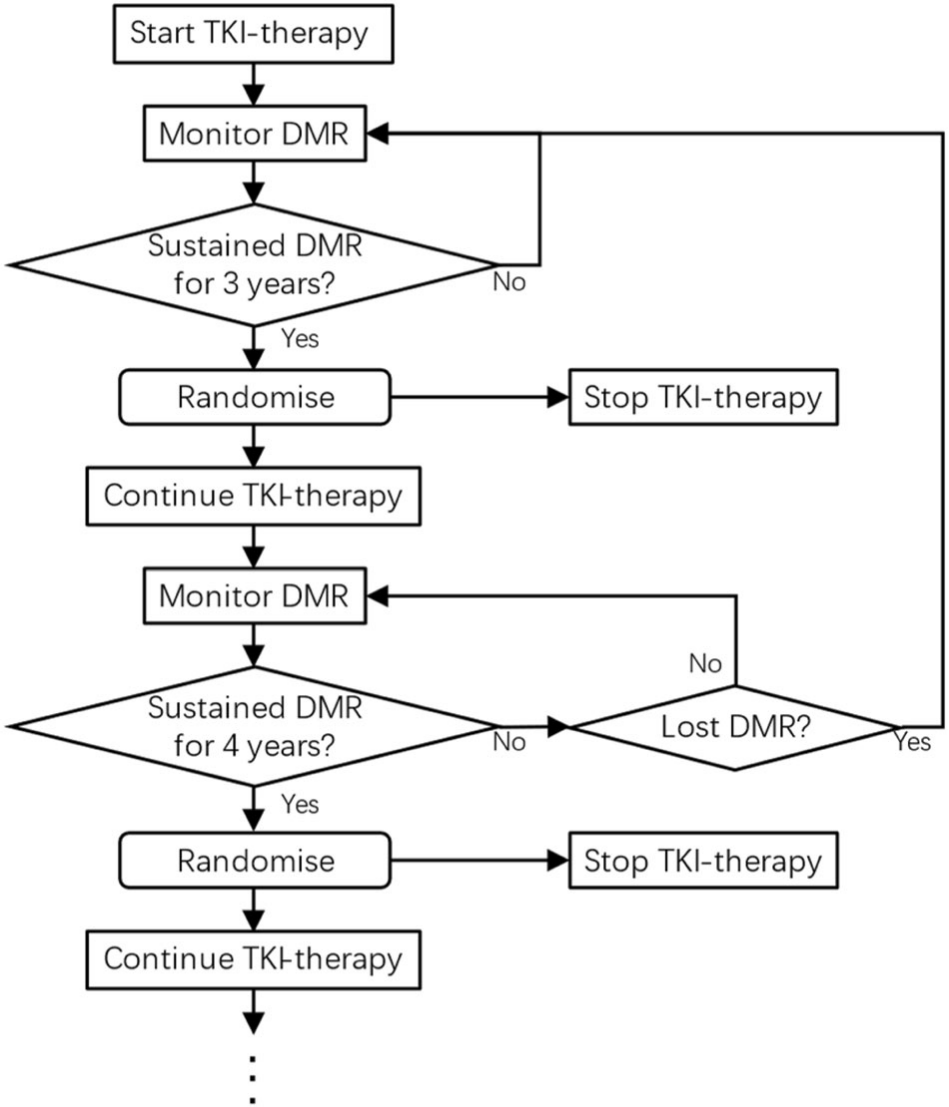
Outline of a study to determine the best duration of deep molecular remission (DMR) to achieve therapy-free remission (TFR).

Yes and no. The EURO-SKI report did not inform us how to treat someone with a DMR on TKI-therapy at a specific time point. A randomized controlled trial, however, might determine the best duration of TKI-therapy by answering this question at the *cohort* level: For people with CML who have sustained DMR for *X* years, should we continue or discontinue TKI? In such a trial everyone is registered at the start of TKI-therapy. At a pre-specified interval, say 3 years, subjects meeting pre-specified criteria (perhaps same as those in the EURO-SKI study) are randomly-assigned to stop TKI-therapy or to continue. At the next pre-specified interval, say 4 years, people still maintaining DMR on TKI-therapy are again randomized to stop or continue and so on. This trial also has limitations but is preferred over the EURO-SKI study-design. For example, some subjects randomized to continue TKI-therapy at 3 years may no longer be eligible to stop at 4 years because of loss of DMR, death (CML-related or not), premature stopping of TKI-therapy (due to an adverse event, cost or other reason(s)), loss to follow-up or withdrawal of consent. Obviously, analyses should be by *intent-to-treat*. Design of such a trial is shown in the Fig. 1.

When everyone with CML starting TKI-therapy is considered only about one-half achieve goal posts (i.e. DMR of various durations) where discontinuing therapy to attempt to achieve TFR is judged appropriate. Moreover, only one-half of these achieve TFR or roughly one-quarter of people with CML. So perhaps the issues we discuss are not of concern to many hematologists. However, given limitations of generalizability of the studies we discussed it’s important to continuously evolve consensus guidelines regarding when to attempt to achieve TFR [15, 16]. We would do well to recall a Dr. Michael Crichton quote: *Historically, the claim of consensus has been the first refuge of scoundrels; it is a way to avoid debate by claiming that the matter is already settled. Whenever you hear the consensus of scientists agrees on something or other, reach for your wallet, because you’re being had. The greatest scientists in history are great precisely because they broke with the consensus*.

## The bottom line

We end our Perspective with these conclusions: 1st, because the EURO-SKI study population is not representative of people with chronic phase CML on TKI-therapy today you should not use the study conclusions to decide whether to continue your patient on TKI-therapy will increase his/her chance of successfully achieving TFR. 2nd, if you know your patient’s duration of DMR since starting first-line imatinib you can use the EURO-SKI data to predict his/her likelihood of achieving TFR but with substantial inaccuracy (error rate >30%). Whether this prediction is useful is debatable given most persons failing a trial of stopping TKI can be re-treated with little or no adverse consequence. Also, some patients would be happy with a 30 percent likelihood of stopping TKI-therapy if there are no adverse consequences. All bets are off with patients starting on first-line TKIs other than imatinib. Good luck!

## Footnotes

1 TFR without CML recurrence and where survival is comparable to the sex-and age-matched population is referred to as *operational* cure.
